# Early treatment with anti-α_4_β_7_ antibody facilitates increased gut macrophage maturity in SIV-infected rhesus macaques

**DOI:** 10.3389/fimmu.2022.1001727

**Published:** 2022-11-01

**Authors:** Samuel D. Johnson, Lindsey A. Knight, Narendra Kumar, Omalla A. Olwenyi, Michellie Thurman, Smriti Mehra, Mahesh Mohan, Siddappa N. Byrareddy

**Affiliations:** ^1^ Department of Pathology and Microbiology, University of Nebraska Medical Center, Omaha, NE, United States; ^2^ Department of Pharmacology and Experimental Neuroscience, University of Nebraska Medical Center, Omaha, NE, United States; ^3^ Southwest National Primate Research Center, Texas Biomedical Research Institute, San Antonio, TX, United States; ^4^ Department of Genetics, Cell Biology and Anatomy, University of Nebraska Medical Center, Omaha, NE, United States; ^5^ Department of Biochemistry and Molecular Biology, University of Nebraska Medical Center, Omaha, NE, United States

**Keywords:** simian immunodeficiency virus (SIV), α_4_β_7_ integrin, microbiome, butyrate, myeloid cells, macrophage maturation, mucosal immunology, viral reservoir

## Abstract

Despite advances in combination antiretroviral therapy (cART), people living with HIV (PLWH) continue to experience gastrointestinal dysfunction. Infusions of anti-α_4_β_7_ monoclonal antibodies (mAbs) have been proposed to increase virologic control during simian immunodeficiency virus (SIV) infection in macaques with mixed results. Recent evidences suggested that therapeutic efficacy of vedolizumab (a humanized anti-α_4_β_7_ mAb), during inflammatory bowel diseases depends on microbiome composition, myeloid cell differentiation, and macrophage phenotype. We tested this hypothesis in SIV-infected, anti-α_4_β_7_ mAb-treated macaques and provide flow cytometric and microscopic evidence that anti-α_4_β_7_ administered to SIV-infected macaques increases the maturity of macrophage phenotypes typically lost in the small intestines during SIV disease progression. Further, this increase in mature macrophage phenotype was associated with tissue viral loads. These phenotypes were also associated with dysbiosis markers in the gut previously identified as predictors of HIV replication and immune activation in PLWH. These findings provide a novel model of anti-α_4_β_7_ efficacy offering new avenues for targeting pathogenic mucosal immune response during HIV/SIV infection.

## Introduction

Human immunodeficiency virus (HIV) infections are associated with significant disruption to gut integrity, including microbial dysbiosis, barrier dysfunction, and resultant chronic immune activation (1, 2). These gut pathologies are similar to the symptomology of inflammatory bowel diseases (IBDs), such as Crohn’s disease (CD) and ulcerative colitis (UC), during which abdominal pain, diarrhea, constipation, fatigue, and mood disturbances are commonly reported (3, 4). Currently, vedolizumab, a humanized anti-α_4_β_7_ integrin monoclonal antibody (mAb) is effectively used to ameliorate IBD symptoms and reduce associated damage by blocking gut homing of inflammatory cells by antagonizing α_4_β_7_ integrin binding to its cognate ligand mucosal addressin cell adhesion molecule 1 (MAdCAM-1) on intestinal endothelium (5). Additionally, differences in α_4_β_7_ integrin surface expression on circulating immune cells have been demonstrated between disease-resistant natural primate hosts of the simian immunodeficiency virus (SIV) that do not progress to AIDS-like disease and non-natural hosts that do progress, often to fatal disease, with the latter having significantly higher mean α_4_β_7_ integrin surface expression (6).

Recently, a primatized anti-α_4_β_7_ mAb reagent was developed that could be repeatedly administered *in vivo* to block the higher levels of α_4_β_7_ integrin expression in disease-progressing rhesus macaques (RMs) (7). Furthermore, anti-α_4_β_7_ mAb was shown to reduce gut viral loads following SIV infection (8, 9). Similarly, anti-α_4_β_7_ therapy was found to reduce gut lymphoid aggregates that could serve as potential HIV-1 reservoirs (10). Despite this reduction, other studies have reported conflicting results suggesting that anti-α_4_β_7_ therapy does not reduce peripheral viral loads/viral reservoirs in SIV-infected rhesus macaques (11–13). Intriguingly, in a clinical trial involving the administration of anti-α_4_β_7_ mAb to HIV-infected individuals revealed that five out of eighteen participants controlled viremia to less than 1,000 copies per mL for the 26 week duration of analytical antiretroviral therapy interruption highlighting the potential utility of anti-α_4_β_7_ therapy towards HIV control (14). Likewise, recent findings noted that anti-HIV broadly neutralizing antibodies-treated RMs that were given anti-α_4_β_7_ therapy delayed viral rebound compared to those treated with broadly neutralizing antibodies alone (15). Because some studies have demonstrated benefits, understanding why certain individuals/macaques fail or succeed in controlling viremia following treatment interruption could provide crucial insights into future designs of HIV/SIV cure studies.

Although vedolizumab was initially developed to reduce pro-inflammatory lymphocyte trafficking to the gut, thereby reducing local tissue damage, several studies have questioned this proposed mechanism (16). Compared to infliximab (anti-TNF-α mAb), vedolizumab is not associated with changes to total, CD4+, CD8+, central memory, or effector memory lamina propria T cells in rectal biopsies of CD and UC patients at week 14, following three mAb infusions (17). Even though infliximab was associated with decreased T-cell recruitment to the gut, it was less efficacious than vedolizumab in measures of both clinical response and remission (17). However, vedolizumab was associated with a decrease in pro-inflammatory M1 and an increase in anti-inflammatory M2 macrophages. These changes were predictive of remission (17). Interestingly, macrophage turnover in the intestines is predictive of accelerated disease progression in SIV-infected macaques (18, 19). Whether the similarities between IBD and HIV/SIV pathogenesis include macrophage recruitment remains poorly characterized.

In addition to the emerging understanding of myeloid cells as potential mediators of IBD, new studies involving the role of the microbiome are providing further insights into IBD pathogenesis. Individuals with IBDs tend to have a decrease in markers of overall microbiome richness, including α-diversity measuring total operational taxonomic units (OTUs) and Shannon diversity index, which incorporates weighting species abundance evenness (20, 21). Additionally, IBD is associated with an increase in the ratio of Bacteroidetes: Firmicutes (B: F), including reductions in Firmicute butyrate-producing bacteria (BPB) like *Roseburia* and *Faecelibacterium* (22–24). Similarly, HIV infection is also associated with dysbiosis with an expansion of the Bacteroidetes genus *Prevotella* and a decrease in BPB (25, 26). The relative abundance of BPB in people living with HIV has been inversely correlated with markers of microbial translocation, immune activation, and vascular inflammation (25). These findings raise the question of whether microbiome composition could further modulate anti-α_4_β_7_ efficacy in SIV infection, possibly contributing to divergent outcomes during therapy.

We characterized gut macrophage maturation dynamics in the duodenum and ascending colon of SIV-infected RMs utilizing both flow cytometry and microscopy techniques and determined tissue viral loads in each compartment prior to and following anti-α_4_β_7_ administration. Additionally, we performed 16S rRNA gene sequencing to determine if microbiome composition impacted these immune dynamics. All macaques were infected with SIVmac251 and CD8-depleted to accelerate pathogenesis as part of a more extensive separate study. As expected, without CD8^+^ cells, the RMs did not control viral replication, reinforcing recent reports that when CD8+ cells are depleted, anti-α_4_β_7_ therapy fails at maintaining viral control during SIV-nef-stop infection (27). However, despite these changes, significant decreases in myeloid cell turnover were seen in the small intestine in the anti-α_4_β_7_ group compared to IgG-treated controls. These changes were associated with alterations in tissue viral loads and markers of dysbiosis in the fecal microbiome, suggesting that myeloid cell dynamics and microbiome composition may be crucial to the efficacy of anti-α_4_β_7_ mAb treatment during SIV infection. These findings provide a new variable for differential experimental outcomes that has until now remained undefined.

## Materials and methods

### Animals and ethics statement

A total of nine outbred Indian-origin rhesus macaques (*Macaca mulatta*) (RMs) with a mean age of 6.1 years old (5.1 to 10.0) were procured from the Yerkes National Primate Center of Emory University, Atlanta, Georgia, the USA and the New Iberia Research Center of University of Louisiana, Lafayette, Louisiana, USA and used in this study as summarized in **Table S1**. All RMs were housed at the Department of Comparative Medicine, University of Nebraska Medical Center (UNMC), Omaha, Nebraska, the USA, in compliance with regulations outlined in the Animal Welfare Act and the Guide for the Care and Use of Laboratory Animals. Animals were pair-housed with visual access to other monkeys in a temperature-controlled (72°F) indoor climate with a 12-hour light/dark cycle. Animals were fed a monkey diet (Purina) twice daily, supplemented with fresh fruits and water being available *ad libitum*. Animals were anesthetized (ketamine 10 mg/kg or telazol 4 mg/kg) prior to all procedures, and meloxicam (0.2 mg/kg) was administered when appropriate. At the end of the study, euthanasia was performed following the guidelines of the American Veterinary Medical Association by a high dose of ketamine-xylazine followed by exsanguination and cardiac perfusion. This study was approved by the UNMC Institutional Animal Care and Use Committee (IACUC) and the Institutional Biosafety Committee (IBC) under protocol #15-102-12-FC entitled “Gut Trafficking Cells in SIV Infection”.

### Study design

Nine RMs were randomly divided into two groups: four in the control group were administered control IgG and five in the experimental group treated with a primatized monoclonal anti-α_4_β_7_ IgG. All monkeys were depleted of CD8+ cells by the administration of a rhesus recombinant IgG1 anti-CD8α mAb (MT807R1) Lot: 100LBRX-3206-023-001 (NIH Nonhuman Primate Reagent Resource) four times spanning several days before and after infection (Day -4: 10 mg/kg subcutaneously and on days -1, 3, and 6 administered 5 mg/kg intravenously). On Day 0, all macaques were intravenously inoculated with 1000 TCID_50_ SIVmac251 (Source: Simian Vaccine Evaluation Unit, NIAID, NIH). During acute infection on Day 12, daily intramuscular administration of a combination of anti-retroviral therapy (cART) (20 mg/kg TFV + 40 mg/kg FTC + 2.5 mg/kg DTG) was initiated and continued until Week 14. On day 15, while the controls were administered 50 mg/kg rhesus IgG1 control antibody rhesus IgG1 mAb (DSPR1) Lot: LH15-35 (NIH Nonhuman Primate Reagent Resource), the experimental group monkeys were administered 50 mg/kg rhesus recombinant IgG1 anti-α_4_β_7_ mAb (A4B7, Lot: LH17-14-NIH Nonhuman Primate Reagent Resource). Antibody infusions were continued every three weeks until week 23 for a total of eight infusions. Blood was collected from the saphenous vein using K2-EDTA vacutainer tubes (Becton, Dickinson, San Diego, CA, USA) for monitoring plasma viral loads. Feces were collected using fecal loops at baseline and on days 14, 98, and 161 and were snap frozen for future analysis. At necropsy, gut tissues were collected, a portion snap frozen, a portion fixed in 10% formalin, and additional tissue used for immune cell isolation (below) for flow cytometry. An illustration of the study design is available in **Figure S1**.

### Intestinal cell isolation

At necropsy, duodenum and ascending colon were collected, washed with DPBS, and dissected into two compartments: 1) epithelium and lamina propria and 2) muscularis externa and serosa. Tissue-specific immune cells were isolated by mincing, digestion with 10,000 U Collagenase, Type 4 (Worthington Biochemical Corporation, Lakewood, NJ, USA; Product #LS004188) and 25 U DNAse I (Roche Diagnostics, Mannheim, Germany; Product #LS004188) in 10 mL DPBS for two hours, filtration through 100 μm and 40 μm sterile cell strainers (Fisherbrand, Hampton, NH, USA; Product #22-363-549 and 22-363-547), and density gradient centrifugation with 60% and 30% Percoll (Cytiva, Uppsala, Sweden; Product #17089101) layers at 2000 rpm for 30 minutes. Isolated cells were washed with DPBS and resuspended in RPMI.

### Flow cytometry

Isolated gut cells were sequentially incubated with Zombie Aqua Fixable Viability Dye (BioLegend, San Diego, CA, USA; Product #423102) to discriminate live/dead cells and incubated with Fc blocker to minimize non-specific binding and then stained with a panel of fluorescent-dye conjugated antibodies (**Table S2**), and then fixed with 1% PFA. Both compensation and fluorescent minus one (FMO) controls were utilized to assist in gating placement. Event acquisition was performed using a Fortessa x450 flow cytometer (B-D, Mountain View, CA). Analysis was performed with FlowJo 10.6.1 software (Treeland, OR). To determine macrophage maturity, a gating strategy developed by Bujko, et al. was utilized (see **Figure S2**) (28).

### Viral loads

Viral loads were determined as has been described previously (29). In brief, plasma was separated from whole blood by centrifugation at 1200 rpm for 20 minutes, and RNA was isolated using a QIAamp viral RNA Mini Kit (Qiagen, Germantown, MD, USA; Product #52906). Frozen gut tissue was lysed using a TissueLyser LT (Qiagen, Germantown, MD, USA; Product #69980), and then DNA and RNA were isolated using an AllPrep DNA/RNA Mini Kit (Qiagen, Germantown, MD, USA; Product #80204) according to manufacturer instructions. Copies of SIVgag DNA were normalized to cell number by quantifying genomic RPP30 as previously described to determine copies/cell (30).

### 16S rRNA sequencing

Fecal samples were later thawed and approximately 100-200 mg of fecal sample was used to isolate DNA using spin column chromatography-based Stool DNA Isolation Kit (Norgen Biotek Corp; Product #27,600) following manufacturer recommendations. DNA was quantified with a GE SimpliNano spectrophotometer and shipped on dry ice to LC Sciences, LLC (Houston, TX, USA) for 16S rRNA sequencing. A library was generated by amplifying the V3 and V4 16S rRNA variable regions and adding sequencing adapters and barcodes after the first cycle. An Illumina cBot system was used to generate clusters for sequencing with an Illumina MiSeq platform. Barcodes were used to separate data, and an in-house script was used to annotate taxa according to RDP, Greengenes, and NCBI 16SMicrobial customized databases for reference. Data output statistics, including clustering into operational taxonomic units (OTU), diversity analysis, species classification, and abundance analysis were performed by LC Sciences, LLC (Houston, TX). FASTQ files were deposited in the NCBI Sequence Read Archive with the BioProject accession number PRJNA870961.

### Immunofluorescence microscopy

Formalin-fixed gut tissues collected at necropsy were embedded in paraffin blocks, cut into 5 µm sections using a microtome (Leica Biosystems, Deer Park, IL, USA; Product #RM2235), and placed on positively charged slides (Avantik, Pine Brook, NJ, USA; Product #SL6332). Tissue sections were deparaffinized with xylene and rehydrated with graded ethanol to water. Antigen retrieval was performed by Decloaking Chamber™ NxGen (Biocare Medical, LLC, Pacheco, CA, USA; Product #DC2012). Tissues were washed three times with water, and a blocking buffer (5% normal goat serum + 1% BSA in PBS) was applied for one hour at room temperature to prevent non-specific binding. Sections were incubated overnight with anti-CD163 and anti-CD206 antibodies (**Table S3**) at 4°C. Tissues were washed with PBS and incubated for one hour with fluorescent secondary antibodies (**Table S3**) at room temperature. Tissues were finally washed five times with PBS, Prolong™ Gold antifade reagent with DAPI (Invitrogen Waltham, MA, USA; Product #P36935) was added, and coverslips were applied. 20X images were captured using a LIONHEART LX Automated Microscope (BioTek, Santa Clara, CA) using Gen5 3.05 software. Co-localization was performed using the JAKoP plug-in for Image J 1.53e (31).

### Statistical analysis

Mann-Whitney tests were implemented when comparing data obtained on the anti-α_4_β_7_ with those obtained on the control groups. Multiple t-tests were utilized when comparing myeloid cell phenotypes. Two-way ANOVA, linear regression, and ratio paired T-tests were performed as specified. All statistical analysis was performed with GraphPad Prism 7 for Mac OS X. Statistical significance was determined as *P* ≤ 0.05.

## Results

### Anti-α_4_β_7_ therapy is associated with increased macrophage maturity in the duodenum

Contrary to early reports, more recent data has increasingly suggested that the mechanism of action for anti-α_4_β_7_ mAbs is more complex than blocking inflammatory lymphocyte infiltration (16). These studies have indicated that myeloid cells, particularly macrophages and DCs, may contribute to the efficacy of vedolizumab (17, 32, 33). To characterize changes in gut myeloid cell differentiation at necropsy, immune cells were isolated from the lamina propria and muscularis externa of the duodenum and ascending colon. The isolated cells were subjected to flow cytometry and a gating strategy was utilized based on a previously described method developed by Bujko, et al., (**Figure S2**) which was focused on the characterization of gut myeloid cells across developmental stages from monocyte-like macrophages (Mf1), intermediate macrophages (Mf2), to mature (Mf3) macrophages (28). Additionally, this strategy identifies muscularis macrophages (Mf4), determining their frequency as a percent of total macrophages.

**Figure 1:**
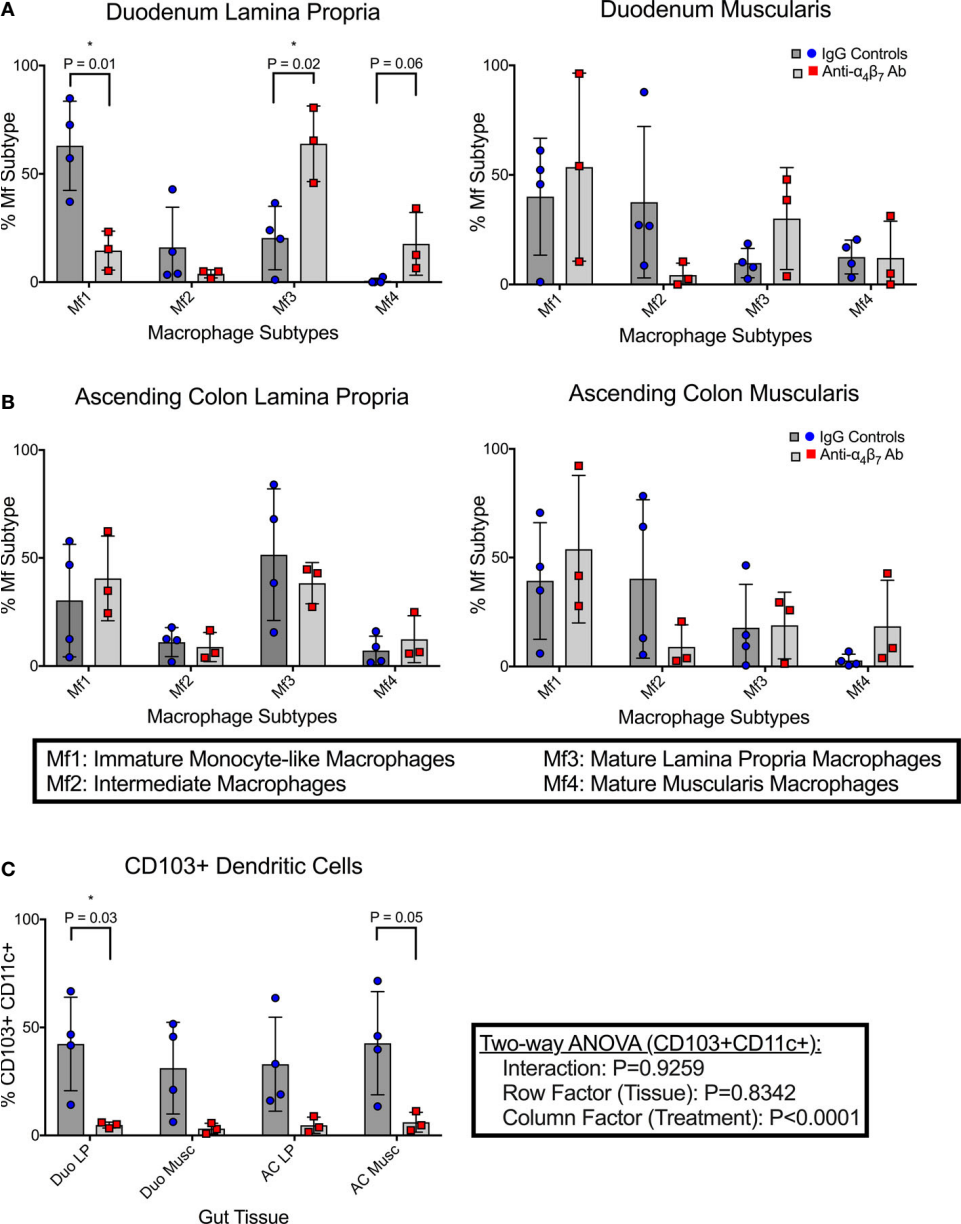
Anti-α_4_β_7_ therapy is associated with reduced myeloid cell turnover in the small intestine. **(A)** Monocyte-like Mf1s were significantly lower in the duodenal lamina propria of anti-α_4_β_7_-treated macaques compared with controls, while mature Mf3 were substantially higher. Similar trends were seen in the muscularis externa. **(B)** This trend was not maintained in the ascending colonic lamina propria. **(C)** CD103 expression on CD11c+ cells was quantified in each tissue type and was significantly lower in the duodenal lamina propria of the α_4_β_7_-treated group * P < 0.05.

Compared to the mean frequency of 63.0% Mf1 macrophages in the duodenum lamina propria of the control group, as seen in **Figure 1A**, the anti-α_4_β_7_ treated group showed a significant reduction (mean 14.6%) (P=0.01). Similarly, there was a trend in reduction of Mf2s in the same tissues with mean values of 16.1% in the control and 3.8% in the anti-α_4_β_7_ mAb treated group (NS) (**Figure 1A**). In contrast, anti-α_4_β_7_-treated RMs had higher mean frequencies of the Mf3 macrophages (63.9%) than 20.4% in the control group (P=0.02). Similarly, there was a trend in increased Mf4s with anti-α_4_β_7_-treatment (17.7% compared to 0.6%, NS), a minority population in the duodenum lamina propria. Similarly, although statistically non-significant, there was a trend in reduced Mf2s and increased Mf3s in the duodenum muscularis externa in the control group as compared to the anti-α_4_β_7_-treatment group.

As mentioned, Bujko, et al. developed a gating strategy for analyzing cells isolated from the small intestine (28). We applied this strategy to cells isolated from the ascending colon (28). No significant differences were found in any of the macrophage subtypes in either the lamina propria or muscularis externa **Figure 1B**. Despite this, some trends existed between the small and large intestinal macrophage subtypes. In all tissue samples, intermediate Mf2s were lower with anti-α_4_β_7_ treatment and, when combined, were significantly different with a mean of 6.5% compared to 26.2% with values from the IgG treatment group (P=0.02). Conversely, in most samples, Mf4s were higher with anti-α_4_β_7_ treatment. Combined, the anti-α_4_β_7_-treatment group had a mean of 15.1% Mf4s compared to 5.8% in the control group (P=0.03). As expected, there was a trend in higher Mf1s and Mf3s in the lamina propria and Mf4s in the muscularis externa.

In addition to pro-inflammatory macrophages, localization of dendritic cells (DCs) to the gut has also been coupled to the expression of the β_7_ integrin (33). This includes CD103+ conventional DCs responsible for imprinting lymphocytes with gut-homing function by releasing retinoic acid (33). The frequencies of CD103+ expressing CD11c+ dendritic cells were quantified in each histological layer (lamina propria and muscularis externa) of both the duodenum and colon (**Figure 1C**). In the duodenum lamina propria, anti-α_4_β_7_ therapy was associated with a significantly lower CD103+ DCs (4.8%) compared to controls (42.4%) (P=0.03). Similar trends were seen in the duodenum muscularis (3.2%, 28.0%; P=0.08), ascending colon lamina propria (4.7%, 33.0%; P=0.08), and ascending colon muscularis (6.1%, 42.7%; P=0.05), with all tissues having lower CD103+ CD11c+ cells in the anti-α_4_β_7_ group. A two-way ANOVA analysis was performed to determine whether the response was dependent on tissue type. No significance was found in the tissue (row factor) or interaction, but the treatment (column factor) was significantly different (P<0.0001), suggesting that the differences were due to treatment and not secondary to the tissue being sampled.

### Anti-α_4_β_7_ therapy is associated with increased colocalization of CD206 with CD163 in gut tissues

**Figure 2:**
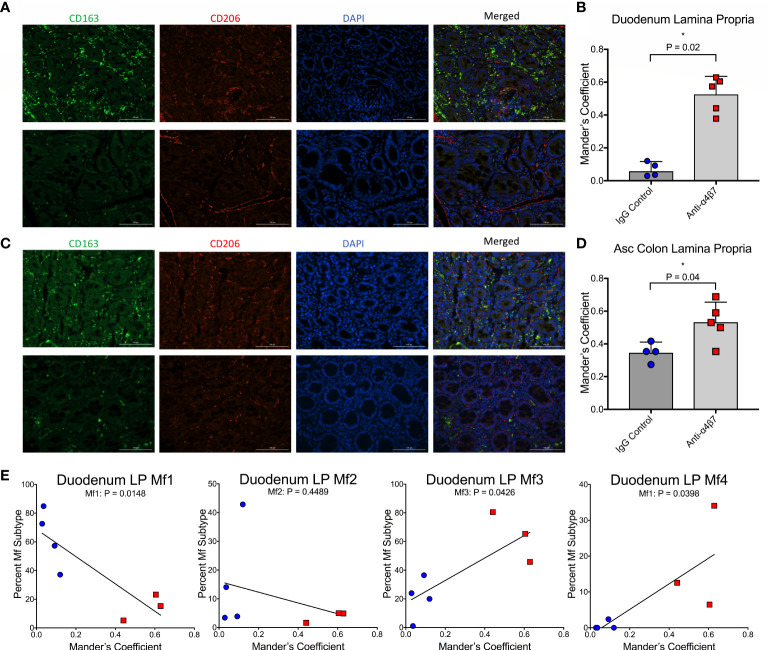
Anti-α_4_β_7_ therapy is associated with increased CD206+ expression on CD163+ cells. **(A)** Immunofluorescence microscopy was used to compare the expression of CD163 and CD206 in duodenum tissue from anti-α_4_β_7_-treated (above) and IgG controls (below). **(B)** Co-localization was quantified with Mander’s coefficient 1, showing the ratio of co-localization of CD206 with CD163. **(C)** Immunofluorescence and **(D)** co-localization were also determined for anti-α_4_β_7_-treated (above) and IgG controls (below) in the ascending colon. **(E)** Linear regression analysis was used to compare co-localization with duodenal lamina propria macrophage subtypes determined by flow cytometry. * P < 0.05.

Previously, it has been suggested that intestinal macrophage turnover is associated with peripheral monocyte turnover and is predictive of disease progression in SIV-infected RMs (19). To demonstrate these associations, a gating strategy determining the ratio of CD163+CD206+ double-positive to CD163+CD206- single-positive cells was utilized in conjunction with BrdU/EdU-labeling and confocal microscopy, with the finding that disease progression is associated with a reduction in the ratio of double-positive to single-positive cells during SIV infection (19). To validate our flow cytometry findings, we utilized a similar strategy to determine the co-localization of CD206 with CD163 in gut tissues (duodenum and ascending colon) of each of the macaques obtained at necropsy (**Figure 2A, C**). In addition to providing further evidence for increased macrophage maturity utilizing different surface markers (Bujko’s strategy does not include either CD163 or CD206), this strategy also provided data for the two anti-α_4_β_7_-treated RMs with missing flow cytometry data (19). When co-localization of CD206 with CD163 was measured in the duodenum, Mander’s coefficient 1 (MC1) was measured providing the ratio of co-localization compared with total CD163 expression. In the IgG-treated controls, the mean MC1 was 0.07 compared with 0.53 in the anti-α_4_β_7_-treated group (P=0.0159) (**Figure 2B**). When the same strategy was performed for the ascending colon, while the control group was 0.35, the anti-α_4_β_7_-treated group was 0.53 (P=0.0397) (**Figure 2D**). In each tissue, the lower MC1 in the control group suggests reduced macrophage maturity compared with the experimental group.

These readings were next compared with macrophage subtype in the duodenum, as expected, Mf1s were negatively associated with MC1 (r=-0.8523, P=0.0148), and Mf3s (r=0.7706, P=0.0426) and Mf4s (r=0.777, P=0.0398) were positively associated with MC1 (**Figure 2E**). While there was a trend in a negative association with Mf2s, this correlation was not significant. This finding is consistent with Bujko, et al’s decision to not use CD206 as a marker for their gating strategy because CD206 is expressed at high levels on Mf2, Mf3, and Mf4 gut macrophages (28).

### Anti-α_4_β_7_ therapy is associated with changes in tissue viral loads

**Figure 3:**
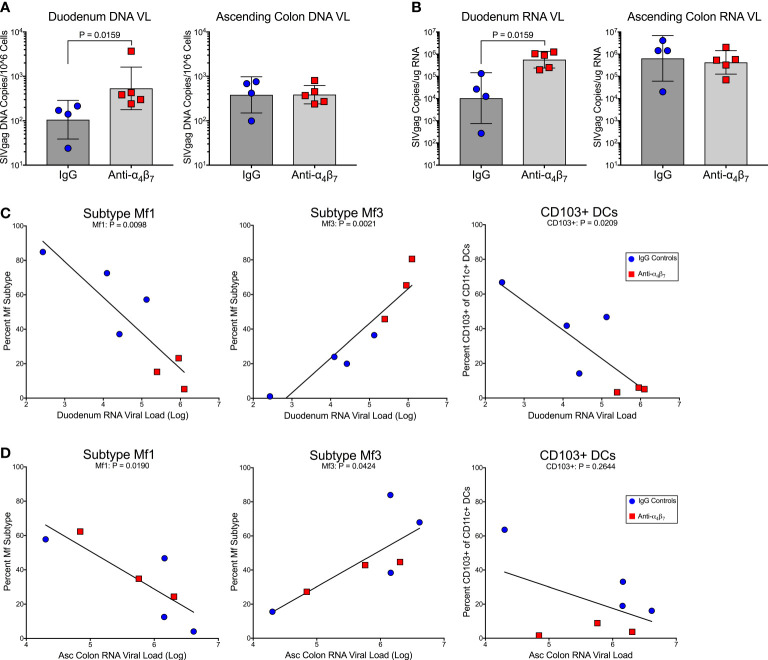
Anti-α_4_β_7_ therapy was associated with higher small intestine tissue viral loads. **(A)** Tissue RNA viral loads were significantly higher in the duodenum of anti-α_4_β_7_-treated macaques compared to controls. **(B)** Tissue DNA viral loads normalized to cell number followed the same trend. **(C)** In the duodenum, viral loads were negatively associated with Mf1s and CD103+ DCs. Viral loads were positively associated with mature Mf3s. **(D)** In the ascending colon, a similar association was found with local viral load and macrophage maturity but not with CD103+ CD11c+ cells.

There were no statistical differences between plasma viral loads during the post-treatment interruption between treated and control animals (**Figure S6**). However, we found significantly higher DNA viral load in the duodenum of anti-α_4_β_7_-treated macaques (geometric mean: 536 copies/10^6^ cells) compared to controls (geometric means 106 copies/10^6^ cells; P=0.02) as determined by copies of SIVgag compared to the host genomic gene RPP30 (**Figure 3A**). No difference in DNA viral loads was found in the ascending colon (387 versus 382 copies/10^6^ cells). A similar trend was seen in RNA viral loads, with the duodenum of the anti-α_4_β_7_ group having a geometric mean of log_10_ 5.8 copies/100 ng RNA compared with to log_10_ 4.0 in controls (**Figure 3B**). Again, no difference was found in the ascending colon (log_10_ 5.6 copies compared to log_10_ 5.8 copies). Next, to determine whether there was a relationship between myeloid cells and viral loads, we performed linear regression analysis on the lamina propria, which were the presumed foci of anti-α_4_β_7_ activity. When both experimental design groups are combined for the duodenum, RNA viral loads were negatively correlated with Mf1s (r= -0.8758, P= 0.0098), positively correlated with Mf3s (r= 0.9332, P=0.0021), and negatively correlated with CD103^+^ DCs (-0.8298, P=0.0209) (**Figure 3C**). Despite few differences between groups in tissue viral load and macrophage maturity phenotype, the two were similarly correlated in the ascending colonic lamina propria with Mf1s negatively (-0.8366, P=0.0190) and Mf3s positively (r=0.771, P=0.0424) correlated (**Figure 3D**). CD103^+^ DCs, though were not significantly associated (r=-0.4899, P=0.2644). Thus, in both the small and large intestines, more mature macrophage phenotypes are associated with viral loads but not necessarily CD103+ DCs. Additionally, the duodenum viral load is associated with MC-1 (r=0.7255, P=0.0270) (**Figure S5**). However, this Pearson’s coefficient is lower than that found when comparing Mf3s (r=0.7255 vs. r=0.9332, respectively), suggesting that fully mature Mf3s, but not all CD163^+^CD206^+^ cells, are closely associated with tissue viral loads.

### Acute SIV infection is associated with acute intestinal dysbiosis

Increasingly, the microbial composition has been recognized as a significant modulator of the inflammatory states of mucosal gut myeloid cells (34). Additionally, specific taxa have been linked with vedolizumab efficacy in IBD patients and lymphocyte activation and viral load during HIV infection (25, 35). To determine whether the microbiome may be influencing the efficacy of anti-α_4_β_7_ during SIV infection, 16S rRNA gene sequencing was performed on DNA isolated from fecal samples obtained at baseline, acute infection (Day 14), during therapy (Day 98), and after cART interruption (Day 161). Using unweighted principal component analysis, it was clear that acute infection was associated with dysbiosis, with animals in each group having microbial composition divergence at Day 14 compared with the other timepoints (**Figure S3a**). Additionally, at the phylum level, non-significant differences were seen in the Bacteroidetes: Firmicutes (B: F) ratio, with the anti-α_4_β_7_-treated group experiencing an increase in Bacteroidetes (B) and a decrease in Firmicutes (F) resulting in an increased B:F ratio at Day 14 (**Figure S3c**). Control animals had the opposite trend suggesting that each group experienced divergent dysbiosis before therapeutic intervention. It should be noted that in the present study, all RMs were CD8-depleted to facilitate rapid viremia and accelerated pathogenesis. Whether CD8 depletion also modulates microbiome composition in SIV-infected macaques remains to be fully elucidated. However, CD8 depletion prevents respiratory syncytial virus-associated dysbiosis in mice, reversing losses in the Firmicute families Lachnospiraceae and Lactobacillaceae (36).

**Figure 4:**
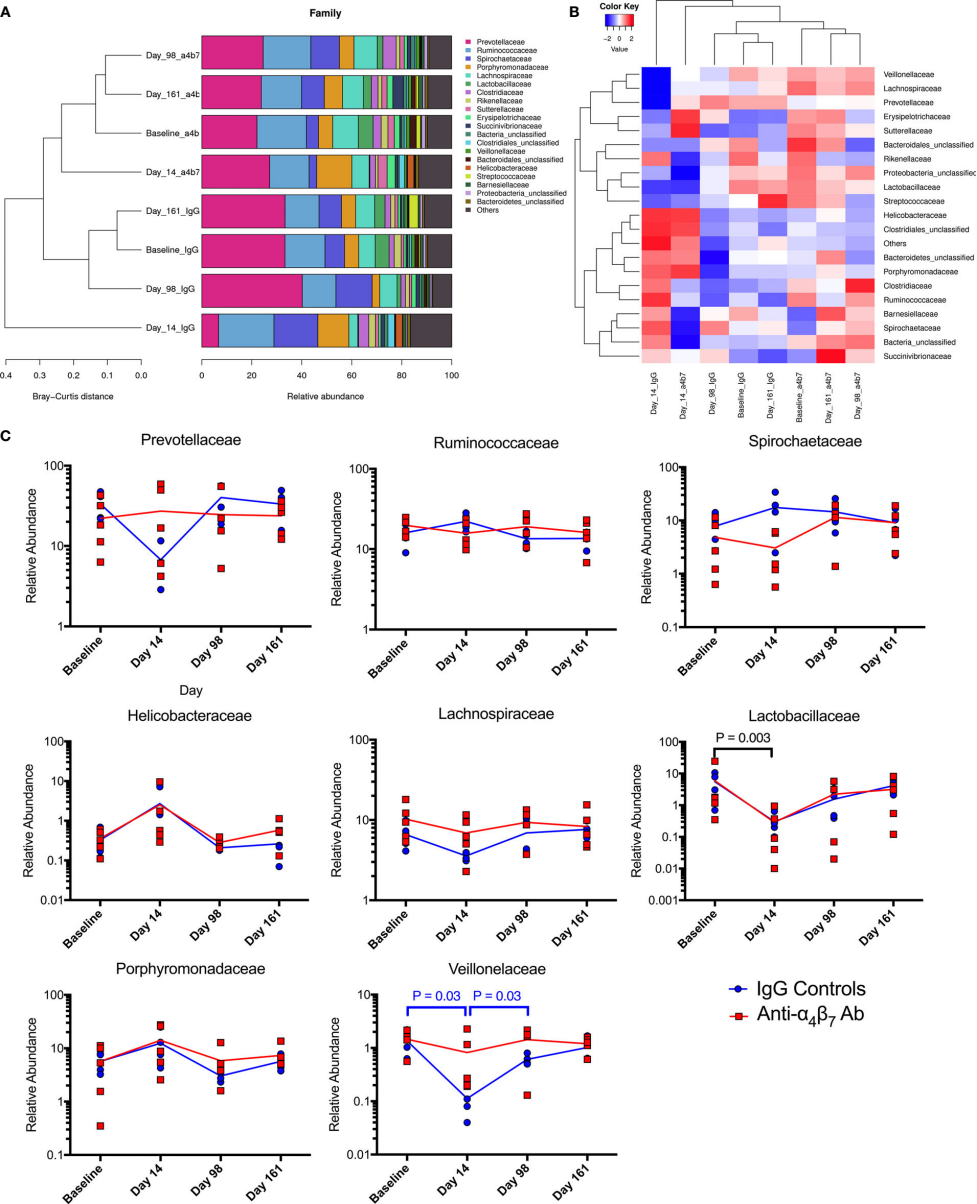
Microbiome family composition is disrupted by acute SIV infection: Both **(A)** stacked grouped abundance and **(B)** heatmap demonstrate acute dysbiosis at Day 14 post-infection where family relative abundance does not group within experimental groups. **(C)** Longitudinal changes to family abundance of Prevotellaceae, Ruminococcaceae, Porphyromonadaceae, Spirochaetaceae, Lachnospiraceae, Lactobacillaceae, Helicobacteraceae, and Veillonelaceae.

For greater granularity in taxonomic changes, we next analyzed the family composition of the microbiome over the time course of the experiment. The data was examined using stacked grouped abundance graphs that demonstrated that samples from Day 14 in each group differed from other timepoints with higher Bray-Curtis dissimilarity in both control and experimental groups (**Figure 4A**). Other time points in each group clustered together with reduced dissimilarity, indicating that each group partially recovered from acute dysbiosis. A heatmap demonstrates similar dynamics, with data on Day 14 samples considered as outliers in each experimental group (**Figure 4B**). Critical families from the above analyses were separated to evaluate specific taxonomic changes better, and longitudinal plots were generated (**Figure 4C**). Prevotellaceae, the most abundant family across all groups, was depleted during acute dysbiosis in the IgG controls, making up only 6.7% in samples from Day 14 compared to 33.3% at baseline. This impact on the relative abundance of Prevotellaceae did not follow a similar trend in the anti-α_4_β_7_-treated experimental group. Conversely, Spirochaetaceae were moderately increased in control IgG group from baseline compared to samples on day 14 (7.8% as compared with 17.6%). None of the above differences were statistically significant between groups due to significant inter-individual variations in microbiome composition. However, both groups had an increase in the relative abundance of the family Helicobacteraceae and a decrease in the family Lactobacillaceae, the latter was significantly different (from 5.8% to 0.3%, P=0.003), with every animal showing a decrease in relative abundance during acute dysbiosis by an average of 88%. Aside from the differences in Prevotellaceae, Lachnospiraceae dynamics were also different between the two groups showing a decrease in the anti-α_4_β_7_-treated group (10.2% at baseline to 8.3%) but an increase in the control group (6.6% to 7.6%). A final significant difference between the two groups was seen in the numbers of the Veillonelaceae family, which was significantly depleted (P=0.03) in the IgG-treated controls after infection but rebounded (P=0.03).

### SIV-associated dysbiosis is partially ameliorated following anti-α_4_β_7_ therapy

**Figure 5:**
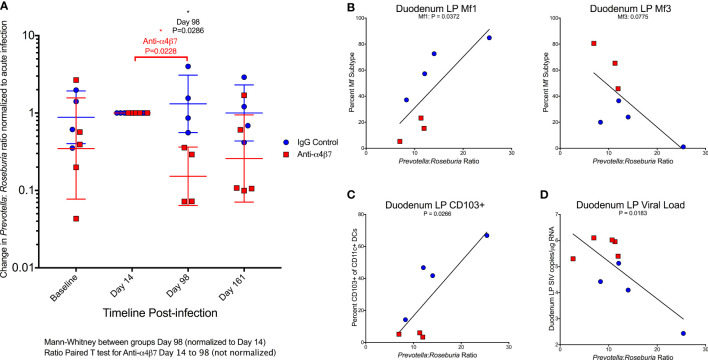
Anti-α_4_β_7_ therapy is associated with a reduction in the *Prevotella*: *Roseburia* ratio. **(A)** The ratio of *Prevotella*: *Roseburia* was calculated and normalized (for visualization and comparison) for the acute infection dysbiosis timepoint (Day 14) for longitudinal fecal samples utilized for 16S rRNA sequencing. Linear regression was performed with this ratio (not normalized) on Day 161 for **(B)** the duodenal lamina propria macrophage maturity phenotypes, **(C)** CD103+ CD11c+ cells, and **(D)** tissue viral loads.

HIV/SIV-associated dysbiosis has been extensively characterized across human populations and non-human primate models. Consistently, infection is associated with an increase in the Bacteroidete: Firmicute ratio in fecal samples. These changes are primarily driven by increases in *Prevotella* and loss of BPB (25, 26, 37, 38). Notably, the BPB genus *Roseburia* has specifically been linked to vedolizumab efficacy, as a marker of HIV-associated dysbiosis (35). The ratio of these two bacteria groups has previously been used as a marker of dysbiosis during HIV infection, significantly higher in PLWH (25). Further, this ratio was positively associated with lymphocyte and colonic dendritic cell activation (25). As previously noted, there was a divergent dysbiosis prior to infection between the IgG and anti-α_4_β_7_ groups. Because of this difference and for improved graphical representation, the *Prevotella*: *Roseburia* ratio was normalized to acute infection (Day 14), the time point at which the microbiome is significantly disrupted and is unable to recover, even with cART (**Figure 5A**). At acute infection, both groups trend to show an increase in the *Prevotella*: *Roseburia* ratio, regardless of whether normalization was performed, consistent with previous observations of acute infection-associated dysbiosis in both HIV and SIV infections (25, 38, 39). One fecal sample on Day 98 was not collected from the anti-α_4_β_7_-treated group. However, in all four animals from which samples were collected, the values were lower following therapy. When normalized to acute infection ratio, a significant difference was found between the IgG- and anti-α_4_β_7_-treated groups (P=0.0286). Even without normalization, the change from acute infection to post-treatment *Prevotella*: *Roseburia* ratio was significant in the anti-α_4_β_7_-treated group compared using a ratio paired T-test that showed a decrease from a geometric mean of 29.7 to 6.5 (P=0.0228).

When the Day 161 *Prevotella*: *Roseburia* ratio was compared with macrophage turnover in the duodenal lamina propria, Mf1s were positively associated (r=0.7833, P=0.0372) with a trend in Mf3s (r=-0.704, P=0.0775) (**Figure 5B**). When the ratio of Mf1:Mf3 was compared with the *Prevotella*: *Roseburia* ratio, the Pearson correlation coefficient was r=0.924 (P=0.0029) (**Figure S4b**). A similar significant association was found in the Mf1:Mf3 ratio in the ascending colon (r=0.8594, P=0.0132) (**Figure S4b**). The *Prevotella*: *Roseburia* ratio was also positively associated with CD103+ CD11c+ cells in the duodenum (r=0.8119, P=0.0266) (**Figure 5C**) and ascending colonic (r=0.9077, P=0.0047) (**Figure S4c**) lamina propria. Finally, the duodenum viral load was also associated with the *Prevotella*: *Roseburia* ratio (r=-0.7565, P=0.0183) (**Figure 5D**). A similar non-significant trend was seen in the ascending colon (r=-0.6104, P=0.0809) (**Figure S4d**). This analysis indicates that increased *Prevotella* and reduced *Roseburia*, two key features of HIV/SIV-associated dysbiosis, are associated with myeloid cell turnover in the duodenum. Further, this dysbiosis was also associated with gut tissue viral loads.

## Discussion

Recent interest in determining the mechanism of vedolizumab action has offered new avenues for understanding HIV/SIV pathogenesis. Contrary to results from earlier studies, clinical samples and murine models of DSS-induced colitis showed an association with an increase in infiltrating monocytes and a decrease in mature macrophages (40). Additionally, β7-integrin-expressing monocyte infiltration exacerbates DSS-induced colitis in RAG2 mice, which lack mature lymphoid cells, emphasizing the role of monocytes, not lymphocytes, in colitis development in these models (32). Mature lamina propria macrophages express reduced CD14 and have a reduced pro-inflammatory response to lipopolysaccharide stimulation from the microbiome (28, 41). However, during IBD, an influx of peripheral CD14+ monocytes and newly-differentiated M1 macrophages capable of microbial pattern recognition enter this niche and release pro-inflammatory cytokines (42). This signaling results in a self-perpetuating cycle of pro-inflammatory macrophage infiltration. These infiltrating monocytes and pro-inflammatory M1 macrophages in the lamina propria are thought to contribute to barrier dysfunction in IBD thought to be secondary to dysfunction of tight junction proteins and the induction of epithelial apoptosis (42). Unlike infliximab, vedolizumab is associated with significant changes in macrophage populations in human patients with CD or UC, not in lymphocyte populations, including a shift from M1 phenotype dominance to M2 (17). Infliximab, though, by blocking TNF-α, may additionally allow a similar increase in anti-inflammatory macrophage phenotypes (43).

SIV infection is associated with a shift in pro-inflammatory monocyte infiltration to gut tissues replacing mature tissue macrophages, similar to the data from IBD patients (44, 45). In RMs, the frequencies of CD163+ macrophages increased four-fold during SIV infection associated with progression to AIDS (46). Additionally, SIV infection is associated with a shift from mature CD163+CD206+ macrophages in the lamina propria to immature CD163+CD206- macrophages, with changes primarily in the small intestine (19). By applying a different flow cytometry gating approach, we provide evidence for lower percent of Mf1 and higher Mf3 macrophages following anti-α_4_β_7_ therapy that is initiated during acute infection, compared with controls. Further, we found that it is the small intestinal lamina propria on which anti-α_4_β_7_ mAb seems to exert the most influence, given the fact that we found differences only in CD206 co-localization with CD163 in the colon using microscopy, but not by flow cytometry, and no differences in the muscularis externa macrophage populations. Additionally, it has been hypothesized that longer-lived macrophages are more likely to serve as viral reservoirs in the tissue (19). Although we did not measure this directly, we found tissue viral loads to be closely associated with Mf3s and negatively associated with Mf1s in both tissues. This was confirmed based on CD206 co-localization with CD163, which also correlates with viral loads in the duodenum. Despite this increase in tissue viral load, it should be noted that natural hosts of SIV experience chronic viremia without progressing to AIDS (47). Results of a recent study in which whole gut tissue transcriptomic data were compared between African green monkeys (AGMs) (*Chlorocebus* spp.; non-progressing natural hosts of SIV) and disease-susceptible RMs following SIV infection demonstrated a significantly higher expression of M2 wound-healing macrophage associated genes in AGMs following SIV infection compared with RM gut tissue which had an increase in pro-inflammatory gene expression (48). Whether anti-α_4_β_7_ facilitates a similar switch to wound-healing remains an additional possibility requiring further investigation.

While anti-α_4_β_7_ alone modulates monocyte trafficking associated with an increase in pro-inflammatory Mf1 macrophages to the lamina propria, we also found the frequency of multiple myeloid cell subsets to be related to the *Prevotella*: *Roseburia* ratio. HIV and SIV are each associated with acute and chronic dysbiosis, even during cART. Prominently, the overall ratio of Bacteroidetes : Firmicutes increases, primarily due to the rise in *Prevotella* at the expense of several Firmicute taxa, a pattern consistent across populations (25, 26, 37, 38, 49, 50). Whether these findings are independent of sexual behavior remains unclear, but similar trends are seen in children and SIV-infected non-human primates (37, 38, 49, 50). In addition to being characteristic of HIV-associated dysbiosis, *Prevotella* has been associated with CD4+ and CD8+ T cell activation in HIV-infected adults (51). *Prevotella* is also negatively related to CD4+ counts in perinatally-infected children and positively associated with plasma IP-10 and soluble CD14 levels, the latter implicating a role in monocyte and/or macrophage activation (37). Further, the inclusion of *Prevotella* in specific pathogen-free mice leads to a significant decrease in the relative abundance of the Firmicutes such as Lachnospiraceae and Ruminococcaceae spp. and exacerbates mucosal inflammation and disease progression in models of colitis in mice suggesting the expansion of *Prevotella* alone may be exacerbating HIV-associated inflammation and dysbiosis (52–54). In contrast to *Prevotella* expansion, several butyrate-producing Firmicute taxa are depleted during HIV pathogenesis, even during cART (25, 26, 37, 38). Most prominently, the relative abundance of colonic *Roseburia* is negatively correlated with plasma viral loads, CD4+ T cell activation, and markers of microbial translocation in PLWH. The colonic ratio of *Prevotella stercorea*: *Roseburia intestinalis* is closely associated with the activation of peripheral CD4+ T cells and colonic DCs, CD4+, and CD8+ T cells in PLWH (25). The short-chain fatty acid butyrate has a pleiotropic role in maintaining gut homeostasis, acting as an energy source for gut epithelial cells, a histone deacetylase inhibitor, and an agonist of GPR41, GPR43, and GPR109A (34, 55). GPR109A agonism specifically facilitates gut macrophages exposed to butyrate to establish their anti-inflammatory phenotype (56). Further, *ex vivo* colonic macrophages cultured with butyrate are imprinted with anti-microbial activity without a concurrent increase in tissue damage (57). Additionally, butyrate-producing bacteria like *Roseburia* have previously been linked to vedolizumab efficacy in IBDs (35), but the results presented herein, we submit, are the first to implicate the microbiome’s role, and the ratio of *Prevotella: Roseburia* specifically, to be closely associated with macrophage phenotype and function following anti-α_4_β_7_ therapy during SIV infection.

The data presented herein represents a study significantly different from previous evaluations of anti-α_4_β_7_ that offers insight into its potential mechanism during SIV infection. Because of these differences, tissue viral loads, in particular, were higher in anti-α_4_β_7_-treated animals compared to controls, while previous reports found the opposite (8, 58). There are several possible reasons for this discrepancy. Computer modeling has shown that anti-α_4_β_7_ mAb’s facilitation of viral clearance was a prominent mechanism in two of the eight animals in the 2016 study (27). Additionally, the macaques in the current study were depleted of CD8+ T and NK cells by administering an anti-CD8α mAb. When performed in the original 2016 cohort following prolonged virologic control, anti-CD8α administration led to rapid viremia (27). Because CD8+ cells have been demonstrated to play a role in anti-α_4_β_7_ efficacy, these findings provide a potential reason for differences in gut tissue viral loads. Finally, diet significantly impacts immune function and viral reservoir (59, 60). Specifically, butyrate produced from the fermentation of dietary fiber can, among other functions, act as an HDAC inhibitor (34). Although HDAC inhibitors have been explored in HIV cure strategies, these studies rarely characterized tissue macrophage reservoirs resistant to cytolysis (61). Whether this mechanism or the immune modulation induced by anti-α_4_β_7_ infusions contributed to differences warrants further investigation, as previously suggested (27). Further investigation is needed to confirm changes in the colon with a larger number of animals to see if the macrophage immunophenotypic differences in the duodenum and its correlation with the *Prevotella : Roseburia* ratio is also present in this tissue.

In addition to immunologic differences, the differences in virus tropism may have also played a role in gut tissue viral load compared with previous studies. Recently, the expression of HIV restriction factor SERINC5 was shown to increase in the process of macrophage differentiation (62). When incorporated into HIV virions, SERINC5 inhibits fusion with target cells, but Nef expression reduces SERINC5 incorporation. Further, HIV-1 ΔNef has a reduced ability to infect mature macrophages. In comparison, infection of HIV-1 ΔNef was non-statistically increased in monocytes (62). If anti-α_4_β_7_ reduces the abundance of monocytes, the lack of functional Nef at infection in the original study may partially explain differences in gut viral loads (8). This is particularly true if longer-lived, mature macrophages serve as reservoirs (19). Even without the nef-stop mutation, SIVmac239 has comparatively lower macrophage tropism than dual tropic SIVmac251 used in this study (63, 64). Additionally, it is known that α_4_β_7_ becomes incorporated into SIV/HIV, and when comparisons of α_4_β_7_ integrin incorporations were made in diverse HIV and SIV strains, it was found that SIVmac251 had the highest levels of incorporation of the 14 total viruses examined (65). Such α_4_β_7_ incorporation into virions has been attributed to increased trafficking to the gut facilitating trans-infection of cells close to MADCAM-1 (65). Regardless of the SIV viral stock used, rapid SIV diversification *in vivo* means that reduced myeloid turnover likely enhances the viral reservoir, thereby increasing tissue viral loads when control monkeys replace macrophages with uninfected monocytes during cART suppression. However, it should be noted that earlier anti-α_4_β_7_ administration studies have been inconsistent, with some unable to replicate viral control and others showing increased time to viral rebound when co-administered with neutralizing antibodies (11, 12, 66, 67). While macrophage turnover is rapid during SIV infection, complete suppression may be necessary prior to anti-α_4_β_7_ therapy to reduce tissue viral loads (19). Future studies need to determine if macrophage turnover and therefore reduced maturity during cART can help limit the tissue macrophage reservoir, thereby explaining discrepancies between our study and previous findings. If true, a delay in initiating anti-α_4_β_7_ administration may improve tissue virologic outcomes.

Although our findings are statistically significant, the interpretation of our data has limitations beyond differences in study design compared with previous similar studies. First, the study utilized for this analysis was designed to test a different hypothesis and was meant to be preliminary. Because of this design, the sample size was small, and two samples were missing for our flow cytometry analysis, thus reducing their statistical power. Next, cART was also interrupted several weeks before necropsy, making translation to IBDs or cART-suppressed PLWH impossible since ongoing viral replication may influence macrophage maturity. Unlike previous studies determining macrophage and monocyte turnover during SIV infection, we did not utilize BrdU to track recently divided cells and instead relied on newly developed flow cytometry techniques and previously validated microscopic markers to determine gut macrophage maturity (19, 28). Finally, the microbiome analysis was performed on fecal samples instead of gut tissue, which are usually only partially correlated (68, 69). Additionally, several factors, including local viral replication, may modulate the microbiome, further complicating interpretation. Therefore, future additional studies incorporating these limitations may address the precise mechanisms behind these observations, and further studies are warranted in this direction.

## Conclusions

**Figure 6:**
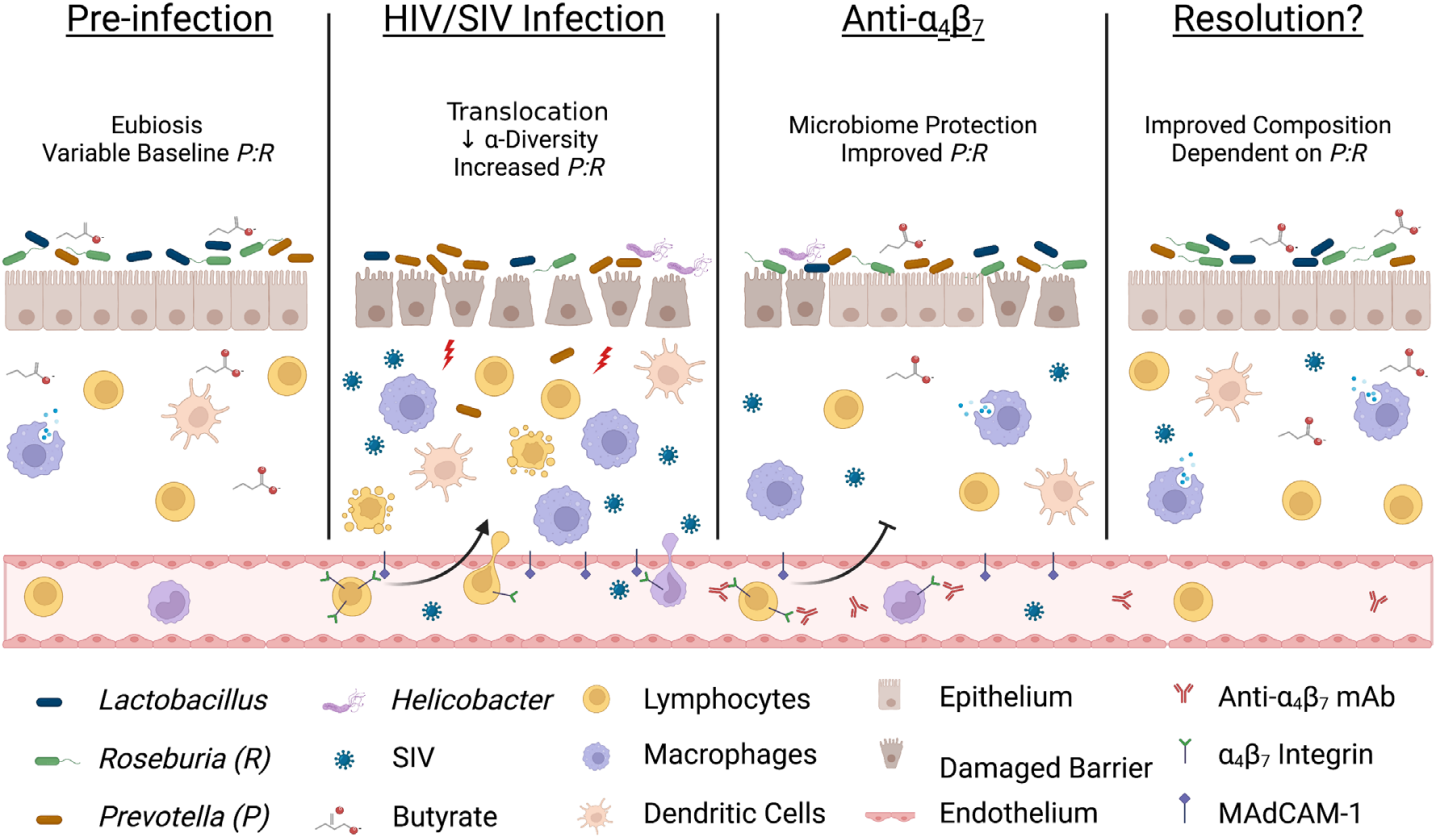
Acute infection dysbiosis is an independent factor in anti-α_4_β_7_ therapy. Several lines of evidence suggest that starting microbial composition can impact the efficacy of anti-α_4_β_7_ therapy. Early during HIV/SIV infection, butyrate-producing bacteria like *Roseburia* are rapidly replaced with *Prevotella* resulting in dysbiosis and an increase in microbial translocation exacerbating pro-inflammatory processes, including trafficking of circulating immune cells. Administration of anti-α_4_β_7_ mAbs facilitates the resolution of inflammation by modulating immune trafficking, including myeloid cells, thereby allowing a reduction in the *Prevotella*: *Roseburia* ratio (*P:R*). However, myeloid cell phenotype, maturation, and function are modulated by butyrate-producing bacteria so if pre-infection levels are low, re-establishment may not be sufficient for efficacy necessitating new approaches to influence gut immune inflammatory milieu Image created with Biorender.com.

The *in vivo* administration of a primatized anti-α_4_β_7_ mAbs during SIV suppression has yielded inconsistent results on virologic control suggesting the involvement of a complex mechanism and possible co-factors that may be responsible for differential efficacy. First, we found that increased macrophage maturity phenotypes were associated with tissue viral loads, a difference from earlier studies that administered anti-α_4_β_7_ after infection. This may indicate that the timing of anti-α_4_β_7_ administration with regard to tissue viral suppression is an essential determinant of efficacy. Second, we found that dysbiosis markers are associated with the relative impact of anti-α_4_β_7_ on macrophage maturity. Low dietary fiber consumption in humans is associated with reduced butyrate production and increased risk of IBD (59, 60), and the relative abundance of BPB has been shown to impact the efficacy of vedolizumab in humans (35). Based on the data presented herein, we propose that important shifts in microbiome composition during HIV/SIV infections like increases in *Prevotella* spp. and depletion of BPB like *Roseburia* spp. may be key independent factors in anti-α_4_β_7_-treated SIV-infected macaques (**Figure 6**). Future studies aiming to modulate gut immune function in HIV/SIV should include assays like 16S rRNA sequencing of colon/fecal samples to characterize microbiome dynamics at baseline, acute dysbiosis, during, and following therapeutic intervention. Further, factors impacting gut microbial diversity and composition, such as animal source, age, co-housing, antibiotic use, and diet, should be considered when interpreting such study results.

## Data availability statement

The original contributions presented in the study are included in the article/**Supplementary Material**. FASTQ files generated during 16S rRNA sequencing are deposited in the NCBI Sequence Read Archive with the BioProject accession number PRJNA870961.

## Ethics statement

The animal study was reviewed and approved by University of Nebraska Medical Center Institutional Animal Care and Use Committee (IACUC).

## Author contributions

SB designed the study and SJ, LK, participated in research design. SJ and LK performed animal protocols. OO designed antibody panel and ran flow cytometer. SJ performed flow cytometry analysis. SJ and LK performed microbiome assays. LK and MT determined viral loads. NK performed microscopy and co-localization quantification and wrote microscopy methods. SJ performed data analysis and wrote the manuscript, which all authors reviewed. SB designed, acquired funding, supervised the entire study and edited the manuscript. MM and SM edited the manuscript, provided funding support and interpretation of the data. All authors contributed to the article and approved the submitted version.

## Funding

National Institutes of Health grant R21MH113455 (SB). National Institutes of Health grant R01 AI129745 (SB). National Institute of Health Grant R01AI134245 (SM & SB). National Institute of Drugs of Abuse Grant R01 DA052845 (MM & SB).

## Acknowledgments

We Thank Dr. Aftab Ansari for critical reading of the manuscript. We thank UNMC comparative medicine Veterinary staff and SB’s current and previous lab members for animal related experimental support **Figures 6**, **S1** were created with Biorender.com.

## Conflict of interest

The authors declare that the research was conducted in the absence of any commercial or financial relationships that could be construed as a potential conflict of interest.

## Publisher’s note

All claims expressed in this article are solely those of the authors and do not necessarily represent those of their affiliated organizations, or those of the publisher, the editors and the reviewers. Any product that may be evaluated in this article, or claim that may be made by its manufacturer, is not guaranteed or endorsed by the publisher.
