# Supplementary material for: Early treatment with anti-α4β7 antibody facilitates increased gut macrophage maturity in SIV-infected rhesus macaques
Source: Front Immunol. 2022 Nov 1;13:1001727. doi: 10.3389/fimmu.2022.1001727 (PMC9664000; doi:10.3389/fimmu.2022.1001727)
Supplement: Supplementary file 1 [file DataSheet_1.docx]

Supplementary Material

# Supplementary Figures


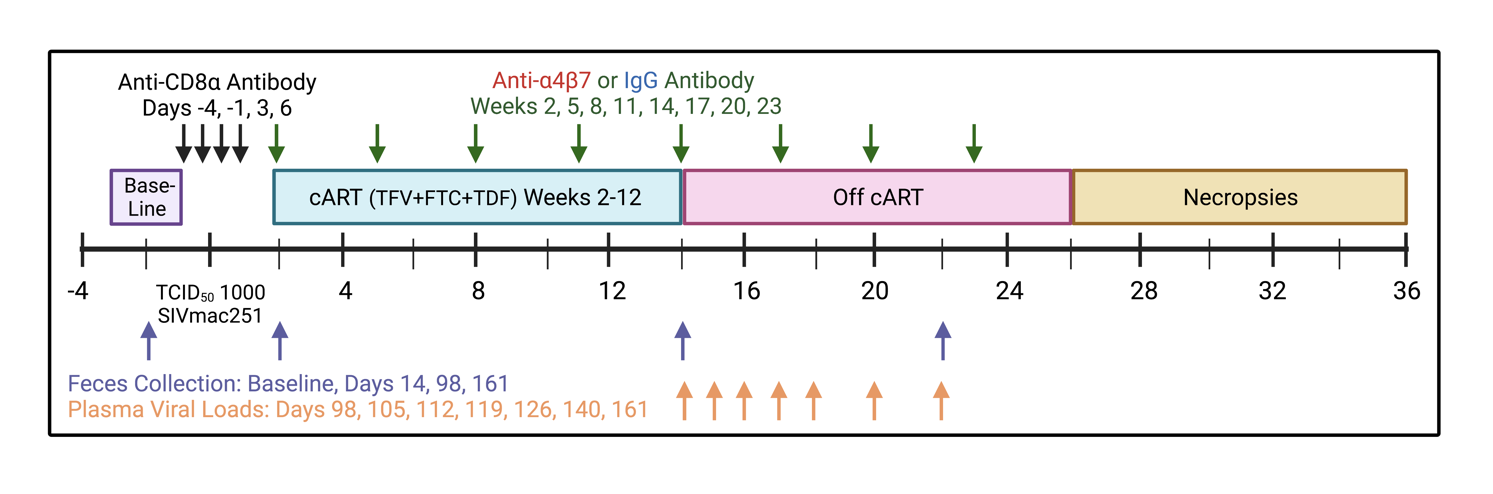


**Supplementary Figure 1. Study Schema**


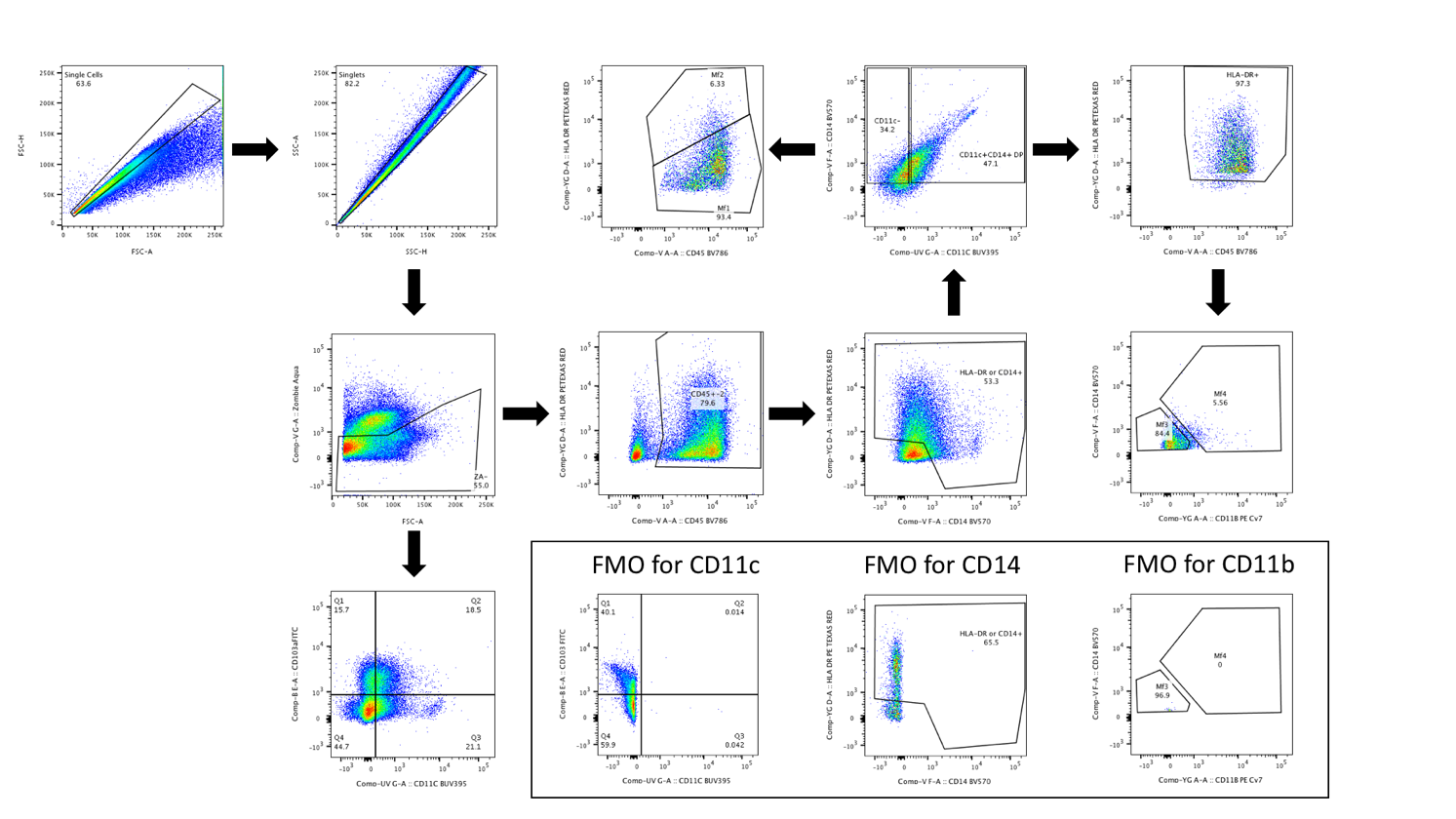


Supplementary Figure 2. Gating Strategy

**
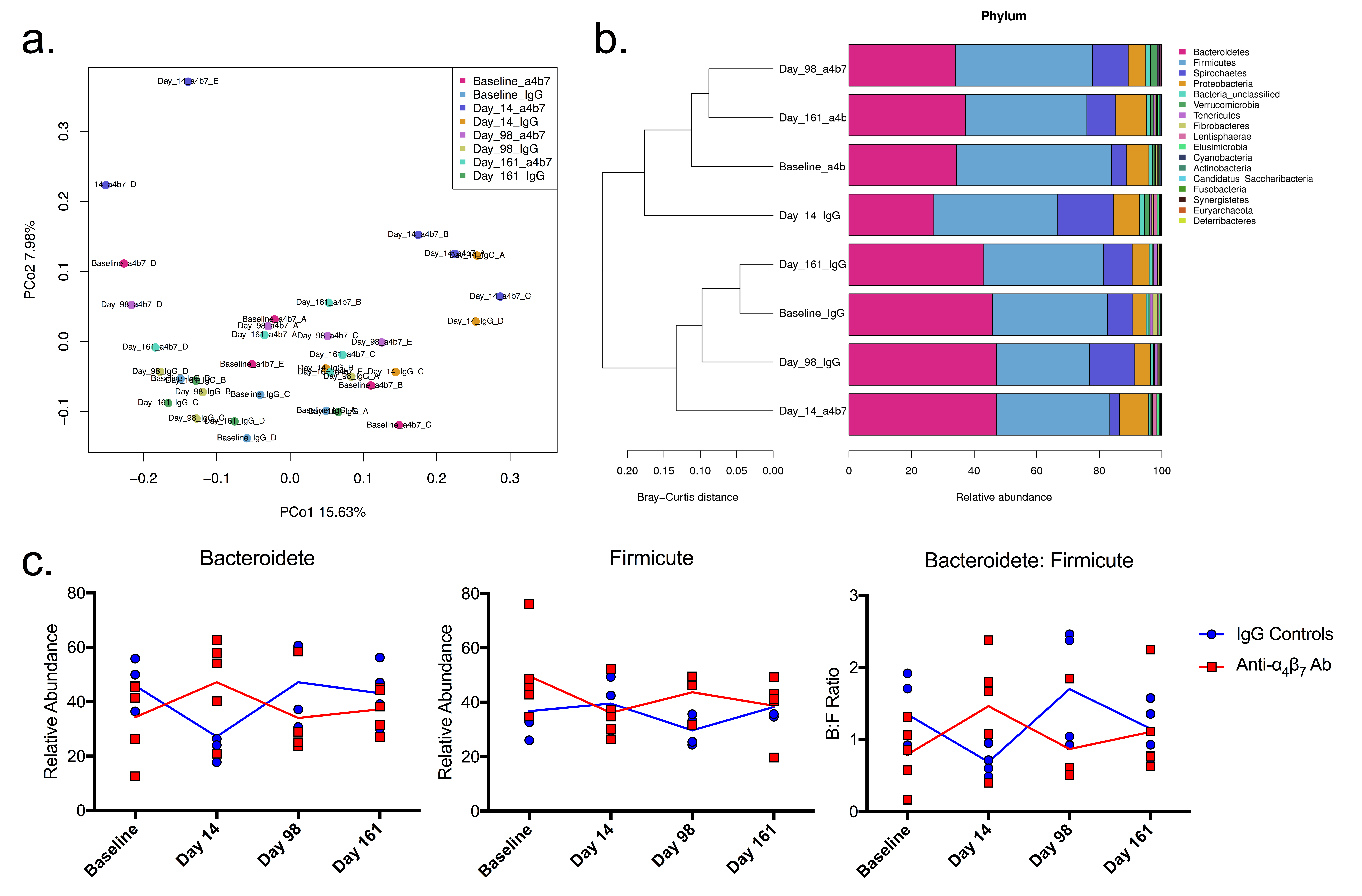
**

**Supplementary Figure 3. Microbiome phyla.** **(A)** Unweighted principal component analysis demonstrates microbial composition divergence at Day 14 of acute infection. **(B)** Grouped phyla abundances are distinct between the experimental conditions and respond differently to SIV infection. **(C)** Bacteroidete and Firmicute dynamics responded differently to acute infection (Day 14) prior for therapy administration and during rebound following therapy initiation (Days 98 and 161).

**
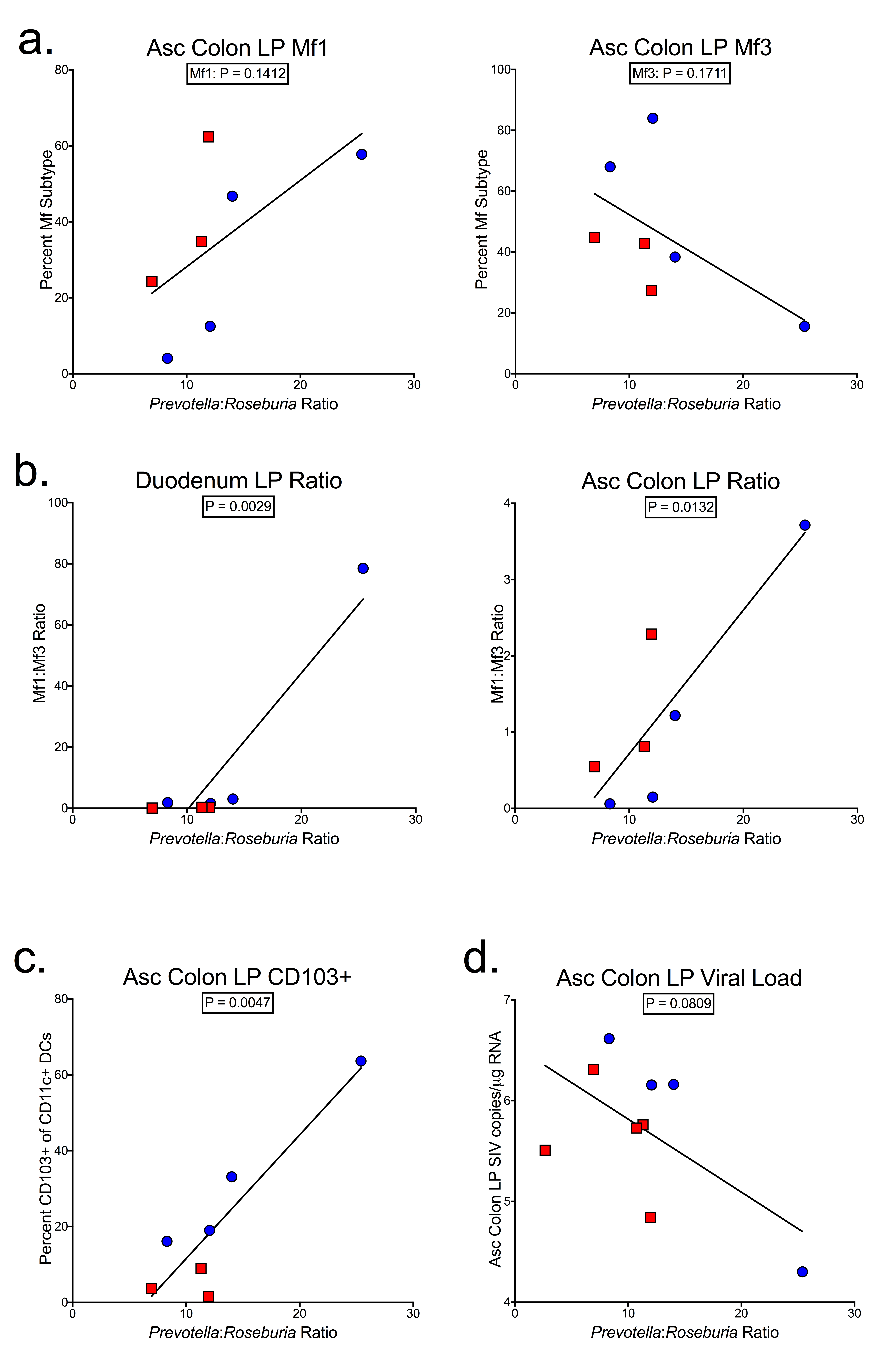
**

**Supplementary Figure 4. Trends in myeloid turnover associations are similar between the duodenum and ascending colon.** The Day 161 *Prevotella*: *Roseburia* ratio is associated with (**A**) macrophage maturity, (**B**) CD103+ CD11c+ cells, and (**C**) tissue viral load in the ascending colon (**D**) The *Prevotella*: *Roseburia* ratio is associated with the Mf1:Mf3 ratio in both the duodenum and ascending colon.





**Supplementary Figure 5: Comparison of Duodenum Viral Loads with Mander’s Coefficient 1.**


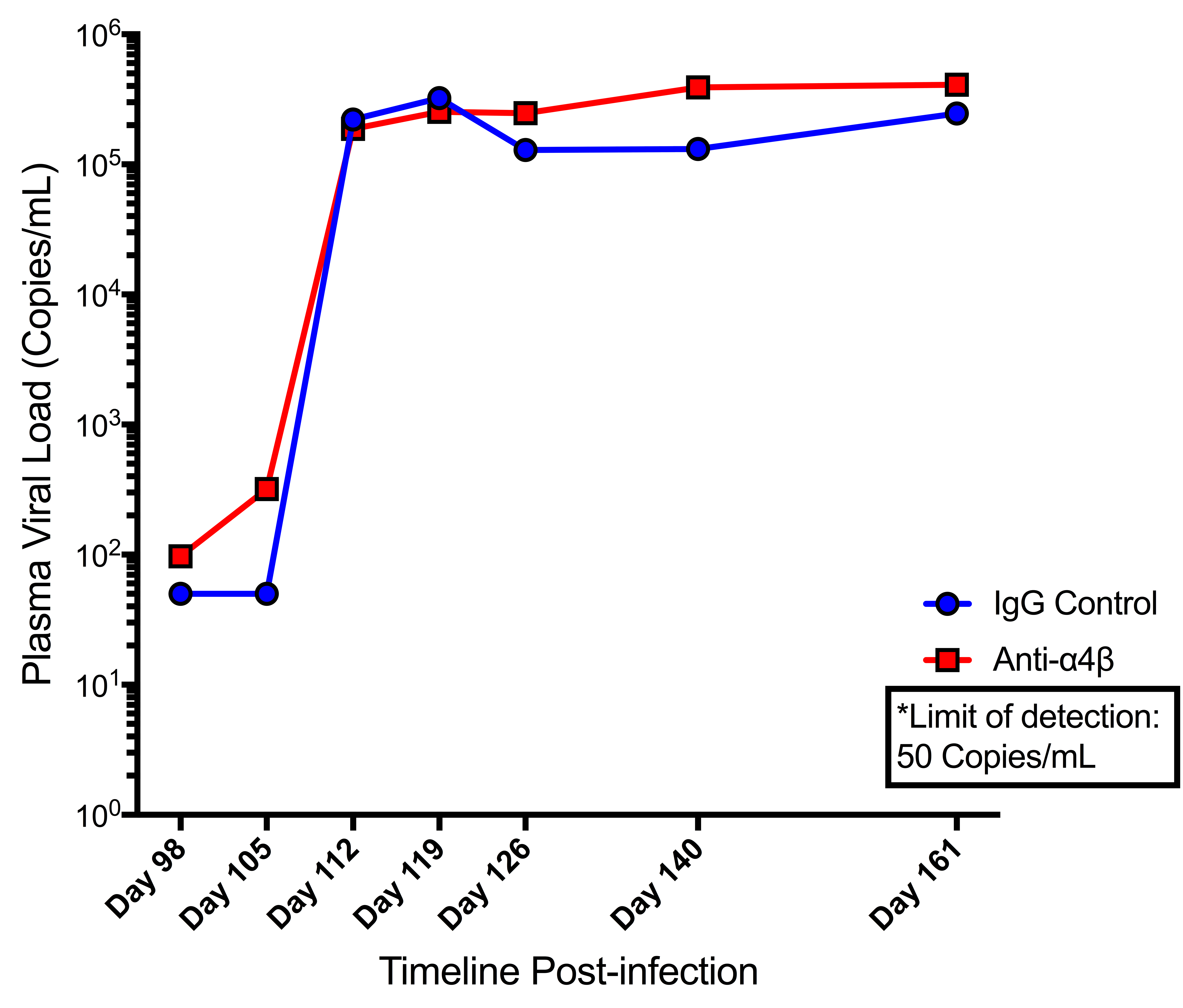


Supplementary Figure 6. Geometric mean of longitudinal plasma viral loads following cART interruption.

# Supplementary Tables

| **Monkey ID** | **DOB** | **Source Primate Center** | **Inoculation Date (SIVmac251)** | | **Weight at Day 0** |
| --- | --- | --- | --- | --- | --- |
| **Controls (IgG Treated)** |  |  |  | |  |
| 13L094 | 5/13/13 | New Iberia | 6/29/18 | | 6.86 |
| A8T007 | 6/17/08 | New Iberia | 6/29/18 | | 7.3 |
| ROg15R | 4/27/12 | Yerkes | 1/19/18 | | 7.58 |
| A12T015 | 6/7/12 | New Iberia | 1/19/18 | | 5.68 |
| **Experimental (anti-a4b7 Treated)** |  |  |  |  | |
| RTh15R | 5/3/12 | Yerkes | 1/24/18 | 9.28 | |
| RVi15R | 5/9/12 | Yerkes | 1/24/18 | 7.42 | |
| RNi15R | 5/25/12 | Yerkes | 1/24/18 | 7.22 | |
| A12T011 | 6/14/12 | New Iberia | 1/24/18 | 5.08 | |
| A12T007 | 5/7/12 | New Iberia | 1/24/18 | 4.6 | |

**Supplementary Table 1. Animal Information.**

| Surface Marker | Fluorochrome | Clone | Company | Titrated Volume (uL) |
| --- | --- | --- | --- | --- |
| CD11b | PE-CY7 | M1/70 | Biolegend | 0.625 |
| CD11c | BUV395 | S-HCL-3 | Biolegend | 0.625 |
| CD14 | BV570 | M5E2 | Biolegend | 1.25 |
| CD45 | BV786 | D058-1283 | BD Horizon | 2 |
| CD103 | FITC | 2G5 | Beckman Coulter | 2.5 |
| HLA-DR | PE Texas Red | MHLDR17 | Life Technologies | 2 |

**Supplementary Table 2. Antibody Panel**

| **Antibody target** | **Clone** | **Source** | **Fluorochrome** | **Vendor** | **Dilution** |
| --- | --- | --- | --- | --- | --- |
| CD163 | EDHu-1 | Mouse | - | GeneTex | 1:200 |
| CD206 | Polyclonal | Rabbit | - | Abcam | 1:200 |
| Anti-mouse IgG | Polyclonal | Goat | Alexa Fluor™ 488 | Invitrogen | 1:2000 |
| Anti-Rabbit IgG | Polyclonal | Goat | Alexa Fluor™ 594 | Invitrogen | 1:2000 |

**Supplementary Table 3: Antibodies used for immunofluorescence staining**
